# Effects of Age on Acute Appetite-Related Responses to Whey-Protein Drinks, Including Energy Intake, Gastric Emptying, Blood Glucose, and Plasma Gut Hormone Concentrations—A Randomized Controlled Trial

**DOI:** 10.3390/nu12041008

**Published:** 2020-04-06

**Authors:** Caroline Giezenaar, Kylie Lange, Trygve Hausken, Karen L. Jones, Michael Horowitz, Ian Chapman, Stijn Soenen

**Affiliations:** 1 Riddet Institute, Massey University, Palmerston North 4474, New Zealand; c.giezenaar@massey.ac.nz; 2 Adelaide Medical School and Centre of Research Excellence (C.R.E.) in Translating Nutritional Science to Good Health, The University of Adelaide, Adelaide, Royal Adelaide Hospital, South-Australia 5000, Australia; kylie.lange@adelaide.edu.au (K.L.); karen.jones@adelaide.edu.au (K.L.J.); michael.horowitz@adelaide.edu.au (M.H.); ian.chapman@adelaide.edu.au (I.C.); 3 Department of Medicine, Haukeland University Hospital, 5021 Bergen, Norway; trygve.hausken@helse-bergen.no

**Keywords:** whey protein, appetite, energy intake, gastric emptying, gut hormones, glucose, aging

## Abstract

Protein-rich supplements are used commonly to increase energy intake in undernourished older people. This study aimed to establish age effects on energy intake, appetite, gastric emptying, blood glucose, and gut hormones in response to protein-rich drinks. In a randomized double-blind, order, 13 older men (age: 75 ± 2 yrs, body mass index (BMI): 26 ± 1 kg/m^2^) and 13 younger (23 ± 1 yrs, 24 ± 1 kg/m^2^) men consumed (i) a control drink (~2 kcal) or drinks (450 mL) containing protein/fat/carbohydrate: (ii) 70 g/0 g/0 g (280 kcal/‘P_280′_), (iii) 14 g/12.4 g/28 g (280 kcal/‘M_280′_), (iv) 70 g/12.4 g/28 g (504 kcal/‘M_504′_), on four separate days. Appetite (visual analog scales), gastric emptying (3D ultrasonography), blood glucose, plasma insulin, ghrelin, cholecystokinin (CCK), glucagon-like peptide-1 (GLP-1) concentrations (0–180 min), and ad-libitum energy intake (180–210 min) were determined. Older men, compared to younger men, had higher fasting glucose and CCK concentrations and lower fasting GLP-1 concentrations (all *p* < 0.05). Energy intake by P_280_ compared to control was less suppressed in older men (increase: 49 ± 42 kcal) than it was in younger men (suppression: 100 ± 54 kcal, *p* = 0.038). After the caloric drinks, the suppression of hunger and the desire to eat, and the stimulation of fullness was less (*p* < 0.05), and the stimulation of plasma GLP-1 was higher (*p* < 0.05) in older men compared to younger men. Gastric emptying, glucose, insulin, ghrelin, and CCK responses were similar between age groups. In conclusion, ageing reduces the responses of caloric drinks on hunger, the desire to eat, fullness, and energy intake, and protein-rich nutrition supplements may be an effective strategy to increase energy intake in undernourished older people.

## 1. Introduction

Protein-rich supplements are commonly used in the older population, and increasingly so due to a growing recognition of the adverse health consequences of nutritional impairment. These impacts include weight loss and a reduction in appendicular muscle mass with associated functional impairment [1], which are symptoms of ‘the anorexia of aging’, defined by the loss of appetite and decrease in energy intake that are associated with aging [2,3]. There is evidence that increased ≥25 g whey protein intake may be beneficial to preserve muscle mass and function in older adults [4,5]. Whey protein was chosen for its high leucine content and rapid digestion, which is suggested to have positive effects on postprandial muscle metabolism [6,7]. There is little data on the effects of protein-rich supplements on energy intake, appetite, and gastrointestinal factors relevant to energy intake, including gastric emptying and appetite-related hormones, e.g., ghrelin, cholecystokinin (CCK), and glucagon-like peptide-1 (GLP-1), in older, compared to younger, adults. We recently reported less suppression of appetite and energy intake by ‘pure’ whey protein drinks and small intestinal infusions of whey in healthy older men compared to younger men [8,9], despite higher CCK concentrations [8] and slower gastric emptying in the older participants—factors that are associated with reduced energy intake in young people [10]. It is suggested that older people have lower sensitivity to regulate their energy intake in response to nutrient intake [9,11,12,13].

In clinical practice, protein-rich nutritional supplements usually also contain carbohydrate and fat. In young adults, high-protein meals may result in slower gastric emptying and increased CCK and GLP-1 responses compared to a high-carbohydrate meal [14], although no differences in gastric emptying were reported in another study including protein, carbohydrate, and fat preloads [15]. The effects of aging on energy intake and underlying gastrointestinal mechanisms after supplements with different macronutrient composition are currently unknown.

The aim of this study was to establish the effect of age on the acute responses to three different protein-rich drinks (i.e., whey protein, with either the addition of fat and carbohydrate, or the equi-energetic substitution of fat and carbohydrate for some of the protein), and a control drink in healthy younger and older men. Ad libitum energy intake at a buffet meal, appetite, gastric emptying rate, blood glucose, plasma insulin, ghrelin, CCK, and GLP-1 concentrations were quantified. We hypothesize that protein-containing drinks will suppress appetite and energy intake less in healthy older compared to younger men, not only when pure protein was administered, but also when protein was administered with other macronutrients.

## 2. Materials and Methods

This was a randomized, double-blind, cross-over study that included 13 healthy younger men (18–35 years) and 13 healthy older men (65 years or older) matched for body weight and body mass indexes (BMI; <30 kg/m^2^). The results for the younger men [16] and older men [17] alone were published previously.

Participants were recruited by advertisement and provided written informed consent prior to their inclusion. They were studied on four occasions, separated by 3–14 days, in random order (30 participants randomized in 1 block with balanced permutations; www.randomization.com) on which they consumed either (i) a control drink (450 mL, ~2 kcal) or iso-palatable and iso-volumetric drinks containing protein/fat/carbohydrate: (ii) 70 g/0 g/0 g (280 kcal/‘P_280′_), (iii) 14 g/12.4 g/28 g (280 kcal/‘M_280′_), or (iv) 70 g/12.4 g/28 g (504 kcal/‘M_504′_). The 70 g whey protein condition (P_280_) was selected as the amount of protein in the pure protein comparator drink as we have previously found that this amount suppresses energy intake, compared to a non-caloric control drink, in younger men (suppression 12 ± 3%), whereas older men showed no suppression (0 ± 8%, *p* < 0.05), in a study using the same study design [17]. The drink-condition effects within each age group, i.e., P_280_ vs. M_280_ vs. M_504_ vs. control, have been described previously [16,17]. 

The study protocol was approved by the Royal Adelaide Hospital Human Research Ethics Committee, and the study was conducted in accordance with the Declaration of Helsinki. The study was registered as a clinical trial with the Australian New Zealand Clinical Trial Registry (www.anzctr.org.au; ACTRN12614000846628).

The exclusion criteria were smoking, alcohol abuse, use of illicit substances, (at risk of) diabetes (HBA_1C_ > 6.0 mmol/L), gallbladder, or pancreatic disease, gastrointestinal surgery (apart from uncomplicated appendectomy), significant gastrointestinal symptoms (abdominal pain, gastro-esophageal reflux, diarrhea, or constipation), use of medications known to potentially affect energy intake, appetite, or gastrointestinal motor function, known lactose intolerance or food allergies, low plasma ferritin levels or blood donation in the 12 weeks prior to the study, and failure to understand the study protocol. 

The study protocol was described in detail in previously published manuscripts [16,17]. In short, after overnight fasting and following drink ingestion, gastric emptying (3D Ultrasonography [18]) and perceptions of appetite and gastrointestinal symptoms (visual analog scale [19]) were determined at 0 (baseline, before drink ingestion), 5, 15, 30, 45, 60, 75, 90, 105, 120, 135, 150, 165, and 180 min. The participants were asked to draw a vertical mark on a 100 mm line in response to questions regarding hunger, desire to eat, prospective food consumption, fullness, nausea, and bloating (for example, ‘How hungry do you feel?’), where 0 mm represented ‘not at all’ and 100 mm represented ‘very much’, based on how they felt at that specific time point. Blood glucose (glucose oxidase method using a portable glucometer; Optium Xceed, Abbott Laboratories, Sydney, NSW, Australia), total plasma insulin (enzyme-linked immunosorbent assay (ELISA) immunoassay), ghrelin (radioimmunoassay; RIA), CCK-8 (RIA), and GLP-1 (RIA) were determined at 0, 5, 15, 30, 45, 60, 90, 120, 150, and 180 min). The homeostatic model assessment (HOMA) index at baseline was used as an indication for insulin-resistance, and was calculated according to the formula: ([fasting insulin concentrations] * [fasting glucose concentrations])/22.5 [20]. Subsequent ad libitum energy intake was determined at a buffet meal [9] (180–210 min).

Based on our previous work, we calculated that 13 participants per age-group would allow for the detection of a difference between groups for suppression in energy intake of 249 kcal (assumed SD = 184 kcal) and T50 of 50 min (assumed SD = 36 min), with power equal to 0.8 and overall alpha equal to 0.05.

SPSS software (version 24; IBM, Armonk, NY, USA) was used to conduct statistical analyses. Independent student t-tests were performed to compare baseline characteristics between age groups. The trapezoidal rule was used to calculate areas under the curve (AUC) for gastric emptying, and AUC’s and change relative to baseline (∆AUC) for blood glucose, plasma hormone concentrations, and perceptions of appetite, in the early phase (from baseline to 60 min), and late phase (60 to 180 min) of gastric emptying. Change relative to baseline was calculated by subtracting the baseline value (at t = 0) from the values at the subsequent time point. Therefore, a negative ∆AUC indicates that the value decreased in response to the ingestion of the relevant study drink, compared to baseline values, whereas a positive value indicates an increase compared to baseline values in response to the study drink. For gastric emptying, glucose, insulin, gut hormone, and appetite AUCs and ∆AUCs, the effects of age, drink condition, and the interaction effect of age by drink condition, were determined using a two-way repeated-measures analysis of variance (ANOVA) model. For gastric emptying, the effects of age, time, and their interaction were determined using a two-way repeated-measures ANOVA model, separately for each condition. When significant interaction effects were present, Bonferroni corrected post hoc tests were performed to determine which specific drink conditions or time points were different between age groups. Independent t-tests were used to determine the effect of age on energy intake suppression. The difference in the suppression of energy intake between the conditions was determined using a paired t-test. Within-subject correlations were determined by using a general linear model with fixed slope and random intercept [21]. Normal distribution of the data was verified for all models. *p* values < 0.05 were considered to be statistically significant. The data are expressed as mean values ± standard error of the mean (SEM) unless otherwise stated. 

## 3. Results

The trial was conducted between August 2014 and February 2016. Thirty people were screened for eligibility, and, of these, 26 were enrolled in the study. A flow diagram is available in Supplementary Figure S1. None of the participants had diabetes or impaired fasting glycemia, although HBA_1C_ was higher in the older men compared to the younger men (older men: 5.4 ± 0.07, younger men: 5.1 ± 0.06; *p* < 0.01). The body weight and BMI of the younger men (age: 23 ± 1 years; body weight: 78 ± 2 kg; height: 1.79 ± 0.02 m; BMI: 24 ± 1 kg/m^2^) and older men (75 ± 2 years; 79 ± 2 kg; 1.75 ± 0.01 m; 26 ± 1 kg/m^2^) were comparable (*p* = 0.63 and *p* = 0.08 respectively), with a trend for higher BMI in older compared to younger men. The study protocol was well tolerated by all participants. In older men, baseline (after overnight fasting) blood glucose and CCK concentrations were modestly higher, and GLP-1 concentrations slightly less, than the younger men (Table 1, all *p* < 0.05). HOMA index at baseline was comparable between the younger men and the older men (average 4 study days, younger men: 1.2 ± 0.2, older men: 1.3 ± 0.5; *p* = 0.16). 

**Figure 1:**
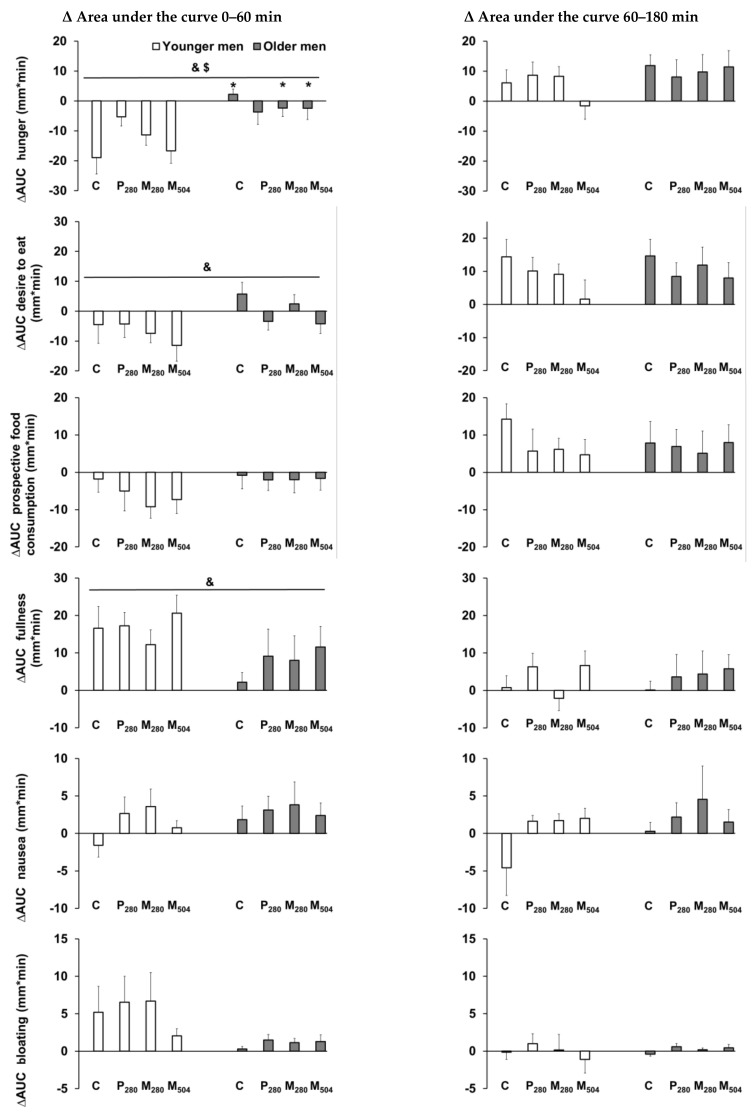
Mean (± standard error of the mean (SEM)) change relative to baseline (after overnight fasting) in the area under the curve (∆AUC) of perceptions of hunger, desire to eat, prospective food consumption, fullness, nausea, and bloating (all mm/min, *n* = 13 healthy young men; *n* = 13 healthy older men) after consumption of (i) a control drink (450 mL, ~2 kcal) or iso-volumetric drinks containing protein/fat/carbohydrate: (ii) 70 g/0 g/0 g (280 kcal/‘P_280′_), (iii) 14 g/12.4 g/28 g (280 kcal/‘M_280′_), or (iv) 70 g/12.4 g/28 g (504 kcal/‘M_504′_). & *p* < 0.05 indicates the overall effect of age. $ *p* < 0.05 indicates the interaction effect of age by drink-condition. * *p* < 0.05 post hoc age by drink-condition interaction effect indicates the conditions for which there was a significant difference between older and younger men.

The interaction effect of age by drink-condition indicated that, in older men, when compared with younger men, ∆AUC hunger was suppressed less by control, M_280_, and M_504_ during the first phase of gastric emptying (0–60 min, interaction effect *p* = 0.048, post hoc analyses all *p* < 0.05; Figure 1). Furthermore, older compared to younger men showed less suppression of overall perceptions of the desire to eat, and less stimulation of overall fullness by drink ingestion (∆AUC_0–60 min_ effect of age all *p* < 0.05).

**Figure 2:**
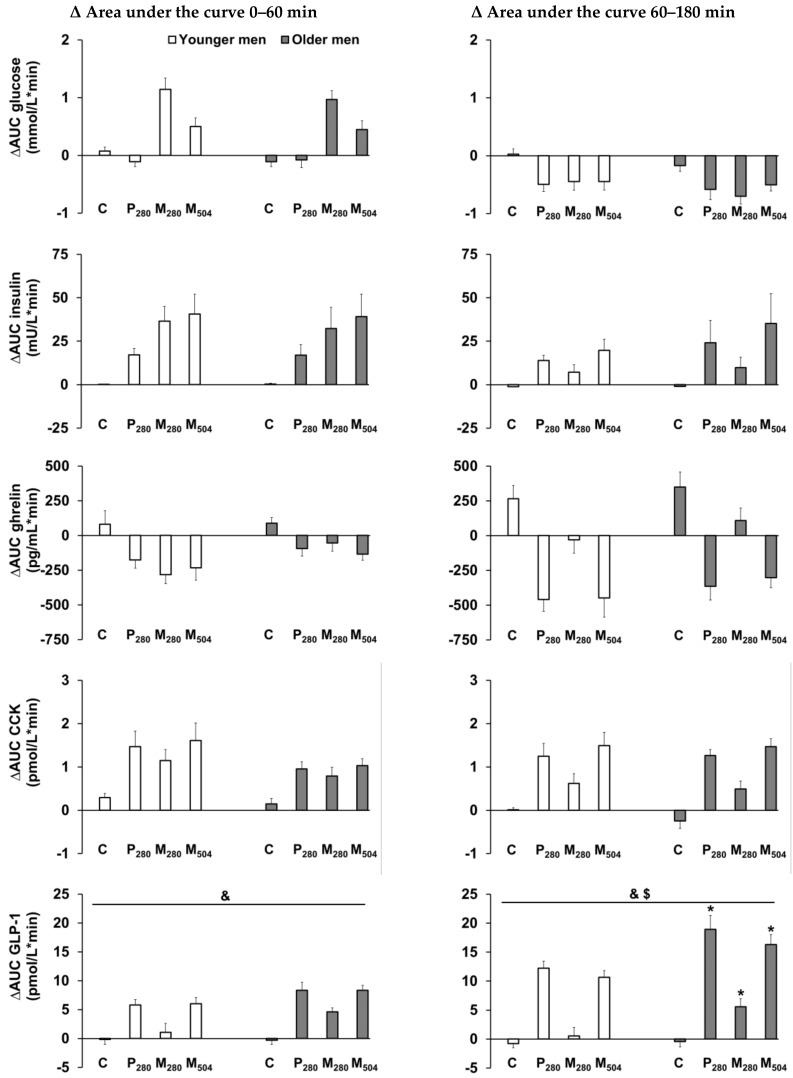
Mean (± standard error of the mean (SEM)) change relative to baseline (after overnight fasting) in the area under the curve (∆AUC) of blood glucose (mmol/L/min) and plasma gut hormone (insulin (mU/L/min), ghrelin (pg/mL/min), cholecystokinin (CCK, pmol/L/min), and glucagon-like peptide-1 (GLP-1, pmol/L/min)) concentrations (*n* = 13 young men; *n* = 13 older men) after the consumption of (i) a control drink (450 mL, ~2 kcal) or iso-volumetric drinks containing protein/fat/carbohydrate: (ii) 70 g/0 g/0 g (280 kcal/‘P_280′_), (iii) 14 g/12.4 g/28 g (280 kcal/‘M_280′_), or (iv) 70 g/12.4 g/28 g (504 kcal/‘M_504′_). & *p* < 0.05 indicates the overall effect of age. $ *p* < 0.05 indicates the interaction effect of age by drink-condition. * *p* < 0.05 post hoc age by drink-condition interaction effect indicates the conditions for which there was a significant difference between older and younger men.

There was an interaction effect of age by drink-condition for the stimulation of ∆AUC GLP-1 concentrations during the late phase of gastric emptying (60–180 min, interaction effect *p* = 0.004; Figure 2). ∆AUC GLP-1 was stimulated more by P_280_, M_280_, and M_504_ in older compared to younger men (post hoc analyses all *p* < 0.05). Together with their lower baseline GLP-1 concentrations, the greater post-nutrient drink increases in GLP-1 concentrations in the older men resulted in no difference between the age groups in the absolute GLP-1 concentrations immediately before the start of the ad libitum test meal (180 min; mean of all study days: younger men: 26.8 ± 2 pmol/L, older men: 26.3 ± 2 pmol/L, effect of age *p* = 0.84).

**Figure 3:**
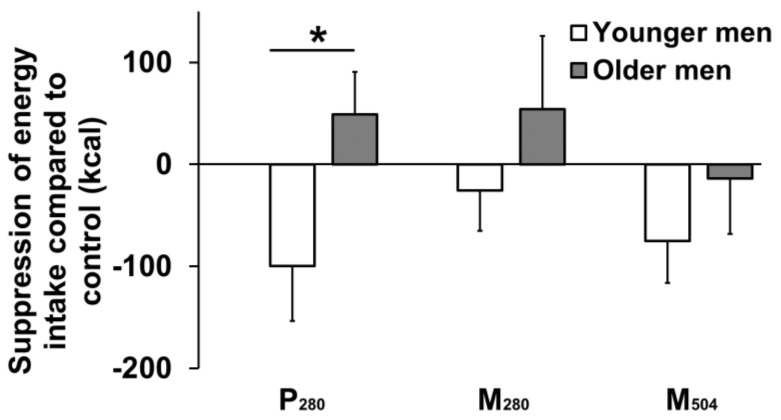
Mean (± standard error of the mean (SEM)) suppression of energy intake at a buffet meal (kcal) after the consumption of caloric drinks (450 mL) containing protein/carbohydrate/fat: (i) 70 g/0 g/0 g (280 kcal/‘P_280′_) (ii) 14 g/28 g/12.4 g (280 kcal/‘M_280′_), (iii)70 g/28 g/12.4 g (504 kcal/‘M_504′_), compared to (iv) control (~2 kcal) in young (*n* = 13, solid bar) and older (*n* = 13, hollow bar) men. * *p* = 0.038, older compared to younger men had less suppression of energy intake by P_280_ compared to control, as assessed with a paired t-test.

The energy intake and macronutrient composition of the test meals is shown in Table 2, and the suppression of that intake relative to control day intake is presented in Figure 3. On the control day, the older participants consumed ~20% less energy than the younger participants, but this difference was not statistically significant (independent t-test, *p* = 0.09). Ad libitum energy intake at the buffet meal after the protein containing drinks was affected by age (Figure 3); there was greater absolute (independent *t*-test, *p* = 0.038), and a trend for percentage change in (independent t-test, *p* = 0.065), the suppression of energy intake (compared to the control day intake) in younger participants compared to older participants. Energy intake after the P_280_ drink was suppressed by 100 ± 54 kcal/−7 ± 4% in the younger men compared to an increase of 49 ± 42 kcal/5 ± 5% in the older men. In older compared to younger men, the mean suppression of energy intake was less after the M_280_ drink, e.g., by 5 ± 68 kcal in the older vs. 74 ± 53 kcal in the younger participants, but this difference was not significant (independent t-test, *p* = 0.43). Energy intake after the M_504_ drink was not different to intake after the P_280_ drink, e.g., in younger men, the mean energy intake was reduced −75 ± 41 kcal compared to control day intake after M_504_ compared to −100 ± 54 kcal after P_280_ (paired t-test, *p* = 0.70). 

**Figure 4:**
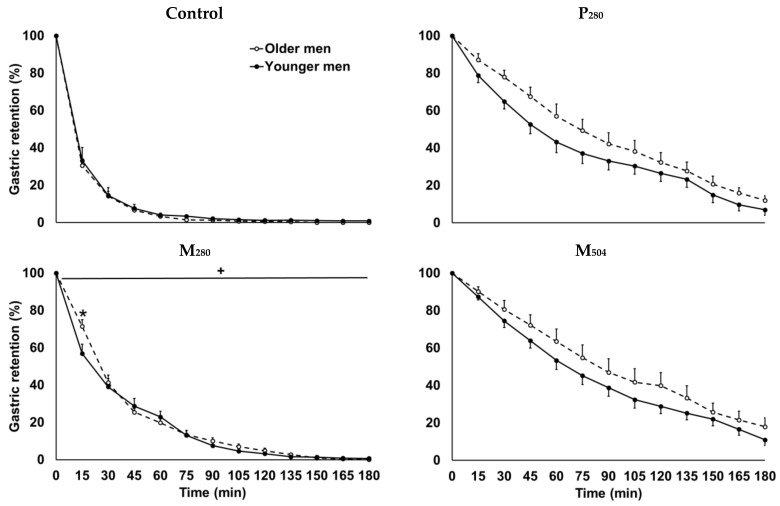
Mean (± standard error of the mean (SEM)) gastric retention (%; *n*= 11 young men, *n* = 9 older men) after consumption of (i) a control drink (450 mL, ~2 kcal) or iso-volumetric drinks containing protein/fat/carbohydrate: (ii) 70 g/0 g/0 g (280 kcal/‘P_280′_), (iii) 14 g/12.4 g/28 g (280 kcal/‘M_280′_), or (iv) 70 g/12.4 g/28 g (504 kcal/‘M_504′_). + *p* < 0.05 indicates the interaction effect of age by time. * *p* < 0.05 post hoc age by time interaction effect indicates the timepoints at which there is a significant difference between younger and older men.

In four older men and two younger men, the quality of the ultrasound stomach images was too low to determine gastric volumes in one or more study conditions, and therefore, all gastric emptying data were removed from statistical analyses for these participants. Gastric emptying showed a comparable linear response after P_280_ and M_504_ and non-linear response after M_280_ and control drink intake in younger and older adults (Figure 4). Gastric retention in the first 60 min after P_280_ tended to be higher in older compared to younger participants (t-test, *p* = 0.05). 

Plasma gut hormone concentrations, and perceptions of appetite and gastrointestinal symptoms were, within participants, related to gastric retention (Table 3). Additionally, energy intake was, within participants, positively related to AUC perceptions of hunger and the desire to eat in the early (0–60min; hunger: r = 0.30, *p* = 0.007; desire to eat: r = 0.33, *p* = 0.003) and late phase (60–180 min; hunger: r = 0.33, *p* = 0.023; desire to eat: r = 0.27, *p* = 0.017) of gastric emptying.

## 4. Discussion

This study establishes that hunger, the desire to eat, fullness, energy intake, and GLP-1 responses to pure whey-protein, and mixed macronutrient, drinks are affected by increasing age in healthy men. In older men, compared to younger men, perceptions of hunger increased less after control and the mixed-macronutrient drinks, overall perceptions of the desire to eat were suppressed less, and overall perceptions of fullness were stimulated less. Energy intake was suppressed less by whey protein, in the older men, compared to the younger men. Gastric emptying, blood glucose, and change relative to baseline in plasma insulin and ghrelin concentrations were comparable between both age groups, while fasting CCK concentrations were higher in older men, and rose to a similar degree in both age groups after the nutrient drinks. In contrast, plasma GLP-1 concentrations were stimulated more in older men compared to younger men. The findings of this study provide additional support for the continued use of protein-rich nutrition supplements to increase energy intake as a means of maintaining or increasing body weight, muscle mass and function, and one’s quality of life.

We, and others, have reported that, in older compared to younger adults, the suppression of appetite and energy intake by nutrient ingestion [9,11,22], including ingestion of 70 g of pure whey protein by healthy men [9], as used in the P_280_ condition in the present study, is less. The observed difference between older and younger men in the extent of the suppression of ad libitum energy intake three hours after the 70 g of whey protein was consumed alone was comparable to that observed in our previous study of healthy men (5), although absolute mean suppression by whey was less in the present study (Δ12%; younger men: −7 ± 4%, older men: +5 ± 5%) than the previous study (Δ12%; younger men: −12 ± 3, older men: 0 ± 8%). The results of this study confirm the lesser suppression of ad libitum energy intake in response to whey protein-rich drinks in older compared to younger men, and also suggest that, in older men, this effect may be evident whether the whey protein is ingested alone or in combination with fat and carbohydrate; in older men, there was no suppression of energy intake by 70 g of whey protein alone in a 280 kcal drink, by 14 g of whey protein combined with fat and carbohydrate in a 280 kcal drink, or by 70 g of whey protein combined with fat and carbohydrate in a 504 kcal drink. The reduced suppression in older compared to young men in hunger after control, M_280_ and M_504_, overall perceptions of the desire to eat, energy intake after P_280_, and the reduced stimulation of overall perceptions of fullness, suggest that older people experience lower sensitivity of the appetite-suppressing effects of mixed macronutrients as well as protein. Older people may have a decreased perception of gastric distension—increased gastric volume achieved by an intragastric balloon was associated with lower suppressive effects on appetite perceptions and energy intake in older compared to younger men [23].

In young adults, ingestion of protein preserves muscle mass, even in a caloric deficit [24], and has greater satiating capacity than dietary carbohydrates or fat [25]. Therefore, young adults commonly consume protein supplements to achieve high protein intake. If protein alone, or in combination with other macronutrients, has the same appetite suppressant effect in older as young adults [26], this could have adverse effects by reducing food intake—the resulting weight loss would be adverse and likely counteract the positive effects of protein on skeletal muscle. The observed age-related reduced responses on hunger, the desire to eat, fullness, and energy intake, suggest accordingly that protein, in doses sufficient to stimulate muscle protein synthesis (i.e., 25 g [4,5,27]), can be administered without suppressing appetite and energy intake in this age group and therefore increase overall energy intake during the day. Literature on energy intake regulation in frail/undernourished older people is limited, however, it is suggested that undernourished older adults show less suppression of energy intake than healthy older adults [28], and, therefore, the results of this study may also apply to older people vulnerable to weight loss.

Non- or low caloric drinks typically empty from the stomach in a non-linear fashion, while high-caloric drinks or solid foods empty in a much slower, mostly linear, pattern following an initial lag phase [29]. In this study, the mixed macronutrient drink containing 504 kcal emptied linearly and predictably much slower than the macronutrient drink containing 280 kcal. We, and others, have reported that the gastric emptying of solid and liquid food is slightly slower in healthy older adults than younger adults, within the expected range of gastric emptying rates (1–4 kcal/min [30,31,32]). In the current study, there was a trend for slower gastric emptying in older than younger men study after P_280_, potentially caused by the greater increase from baseline of GLP-1 concentrations in the older men, which is known to slow gastric emptying by inhibiting gastrointestinal motility and gastric acid secretion [33].

Our findings are consistent with earlier reports presenting higher fasting blood glucose in older compared to younger adults [34,35]. Although it is well established that aging is associated with impaired insulin sensitivity [36], potentially caused by a decrease in lean mass [37], we found similar values for the HOMA index at baseline, and postprandial glucose or insulin concentrations for younger and older men. As the older participants in this study were very healthy and active, and were shown to have normal fasting glycemia at screening, this could explain why glucose regulation did not appear to be impaired with increased age in the present study.

At baseline, CCK concentrations were higher, and GLP-1 concentrations were lower, in older compared to younger men. These data are consistent with previous reports from our department in which older men showed increased fasting CCK concentrations compared to younger men [8,38]. 

Plasma CCK concentrations after an overnight fast were higher in the older than younger men, as previously reported [38], with similar postprandial increases in CCK response to the drinks in both age groups. CCK is an anorexigenic hormone that suppresses hunger and food intake [39]. Older compared to younger adults have at least maintained, and possibly increased, sensitivity to the satiating effects of exogenously administered CCK [40]. Plasma GLP-1 concentrations were lower at baseline in the older men compared to the younger men but increased more in the older men compared to the younger men after the nutrient drinks, as previously reported [41,42]. A recently published longitudinal study measured GLP-1 concentrations within the same healthy older participants ~6 years apart, and found decreased fasting GLP-1 at the follow up compared to the initial study [43]. Similar to CCK, GLP-1 also has satiating effects [39]; however, the sensitivity to GLP-1 in the older population has not been evaluated. If anything, the increased CCK and GLP-1 responses in older participants may have been expected to cause a greater, not reduced, suppression of food intake, and, as such, changes in the physiology of gut hormones with aging do not provide a mechanistic explanation of the reduced suppression of energy intake in the older men.

The limitations of the study included the modest subject numbers and measurement of blood glucose concentrations with a portable bed-side monitor. Nevertheless, the bias in glucose measurement would be consistent across all samples, and the results are clear-cut. Although the researchers used lime cordial to match the study drinks for taste and palatability, the participants’ perception of palatability and taste was not measured. Moreover, habitual energy intake was not assessed. We only investigated the effects of whey protein, and the results may be different for other proteins, as there is evidence that protein source can affect postprandial gut hormone, amino acid profiles, and appetite [44]. Only male participants were included, as men have been shown to be more likely to show the suppression of energy intake than women [45], although this difference was not found between older men and women [46]. Still, the current findings may not apply to older women, or adults aged outside of the age range of the participants included in this study. The effects of this study should be confirmed in an undernourished group of older adults, as this population is the most likely to use and benefit from nutritional supplements.

## 5. Conclusions

In conclusion, this study establishes that increasing age is associated with a reduction in the ability of orally ingested mixed-macronutrient drinks to suppress hunger and the desire to eat and stimulate fullness, and the ability of protein-drinks to suppress energy intake. The use of protein-rich nutrition supplementation by older people to preserve muscle mass and function is, therefore, unlikely to suppress energy intake (and the inherent potential for harmful weight loss), although longer term studies in a clinically relevant population are required to further define such effects.

## Figures and Tables

**Table 1:** Mean baseline concentrations of blood glucose, plasma insulin, ghrelin, cholecystokinin (CCK), and glucagon-like peptide-1 (GLP-1), perceptions of appetite, and gastrointestinal symptoms in younger and older men across the 4 study days

Baseline Values	Younger Men (*n* = 13)	Older Men (*n* = 13)	Age Effect (*p* value)
Blood glucose (mmol/L)	5.3 ± 0.1	5.7 ± 0.1	0.003
Plasma insulin (mU/L)	4.9 ± 1	5.2 ± 2	0.93
Plasma ghrelin (pg/mL)	1779 ± 236	1659 ± 165	0.67
Plasma CCK (pmol/L)	1.3 ± 0.1	2.0 ± 0.2	0.008
Plasma GLP-1 (pmol/L)	20 ± 2	15 ± 1	0.017
Hunger (mm)	46 ± 10	31 ± 13	0.34
Desire to eat (mm)	50 ± 6	30 ± 12	0.26
Prospective food consumption (mm)	56 ± 6	46 ± 14	0.73
Fullness (mm)	4 ± 1	2 ± 1	0.76
Nausea (mm)	6 ± 2	3 ± 1	0.08
Bloating (mm)	6 ± 2	3 ± 1	0.09
Gastric volume (mL)	37 ± 3	33 ± 4	0.31

Baseline values (mean of 4 study days, mean ± SEM) of blood glucose (mmol/L), and plasma insulin, ghrelin, CCK and GLP-1 concentrations, and perceptions of hunger (mm), desire to eat (mm), prospective food consumption (mm), fullness (mm), nausea (mm) and bloating (mm), and gastric volume (mL) in younger (*n* = 13) and older (*n* = 13) healthy men. The effect of age was assessed using an independent t-test.

**Table 2:** Energy, protein, fat, and carbohydrate intake in younger and older men.

	Younger Men (*n* = 13)	Older Men (*n* = 13)	Age Effect (*p* value)
**Control day**			
Energy intake (kcal)	1218 ± 109	972 ± 87	0.09
Protein (g)	21 ± 1	20 ± 1	0.28
Fat (g)	32 ± 2	30 ± 1	0.36
Carbohydrates (g)	47 ± 3	52 ± 2	0.13
**P_280_**			
Energy intake (kcal)	1118 ± 105	1020 ± 95	0.50
Protein (g)	21 ± 1	20 ± 1	0.24
Fat (g)	32 ± 2	29 ± 1	0.10
Carbohydrates (g)	47 ± 3	52 ± 2	0.34
**M_280_**			
Energy intake (kcal)	1193 ± 113	1026 ± 76	0.23
Protein (g)	21 ± 1	20 ± 1	0.42
Fat (g)	32 ± 2	31 ± 1	0.48
Carbohydrates (g)	46 ± 3	51 ± 2	0.18
**M_504_**			
Energy intake (kcal)	1143 ± 112	957 ± 76	0.18
Protein (g)	21 ± 1	21 ± 1	0.88
Fat (g)	32 ± 2	29 ± 1	0.22
Carbohydrates (g)	47 ± 3	52 ± 2	0.20

Mean values (± SEM) of energy (kcal), protein (g), fat (g), and carbohydrate (g) intakes in younger (n = 13) and older (n = 13) healthy men after the consumption of drinks (450 mL) containing protein/carbohydrate/fat: (i) 14 g/28 g/12.4 g (280 kcal/‘M_280_’), (ii) 70 g/28 g/12.4 g (504 kcal/‘M_504_’), (iii) 70 g/0 g/0 g (280 kcal/‘P_280_’), or (iv) control (~2 kcal). The age effect was assessed with an independent t-test.

**Table 3:** Within-subject correlations between the AUC_0–60 min_ and AUC_60–180 min_ of gastric retention, and gut hormones and appetite.

	Combined		Younger men		Older men	
	r	*p*	r	*p*	r	*p*
**AUC_0–60 min_**						
Glucose	0.06	0.63	−0.01	0.96	0.14	0.46
Insulin	0.38	0.003	0.52	0.002		
Ghrelin	−0.31	0.014	−0.42	0.015		
CCK	0.61	<0.001	0.65	<0.001	0.65	<0.001
GLP−1	0.68	<0.001	0.57	<0.001	0.79	<0.001
Hunger	−0.27	0.039	−0.39	<0.001	0.02	0.91
Desire to eat	−0.26	0.039	−0.41	0.015	0.11	0.60
Prospective food consumption	−0.24	0.056	−0.40	0.019	0.15	0.45
Fullness	0.26	0.043	0.28	0.11	0.24	0.22
Nausea	−0.10	0.44	−0.14	0.42	−0.03	0.86
Bloating	−0.25	0.053	−0.31	0.07	−0.28	0.16
**AUC_60–180 min_**						
Glucose	−0.13	0.33	−0.31	0.079	−0.02	0.93
Insulin	0.46	<0.001	0.76	<0.001	0.44	0.019
Ghrelin	−0.70	<0.001	−0.74	<0.001	−0.72	<0.001
CCK	0.71	<0.001	0.75	<0.001	0.72	<0.001
GLP−1	0.71	<0.001	0.73	<0.001	0.70	<0.001
Hunger	−0.04	0.76	−0.23	0.19	0.07	0.73
Desire to eat	0.09	0.48	−0.24	0.18	0.08	0.69
Prospective food consumption	0.02	0.90	−0.15	0.40	0.16	0.41
Fullness	−0.05	0.72	−0.04	0.82	0.15	0.45
Nausea	−0.14	0.26	−0.27	0.13	−0.12	0.56
Bloating	−0.27	0.039	0.45	0.007	−0.14	0.49

r and *p* values of *within-subject* correlations between area under the curve (AUC) early (0–60 min) and late (60–180 min) phase gastric retention (%; *n* = 11 for younger men, *n* = 9 for older men), plasma insulin (mU/L), ghrelin (pg/mL), cholecystokinin (CCK, pmol/L), and glucagon-like polypeptide-1 (GLP-1, pmol/L) concentrations, and hunger (mm), desire to eat (mm), prospective food consumption (mm), fullness (mm), and bloating (mm) in healthy younger and older men (*n* = 13 younger men, *n* = 13 older men). Within-subject correlations were determined by a general linear model with fixed slope and random intercept.

## Supplementary Materials

The following are available online at https://www.mdpi.com/2072-6643/12/4/1008/s1, Figure S1: Flow diagram.

## References

[1] Bauer J., Biolo G., Cederholm T., Cesari M., Cruz-Jentoft A.J., Morley J.E., Phillips S., Sieber C., Stehle P., Teta D. (2013). Evidence-based recommendations for optimal dietary protein intake in older people: a position paper from the PROT-AGE Study Group. J. Am. Med. Dir. Assoc..

[2] Morley J.E., Silver A.J. (1988). Anorexia in the elderly. Neurobiol. Aging.

[3] Soenen S., Chapman I.M. (2013). Body weight, anorexia, and undernutrition in older people. J. Am. Med. Dir. Assoc..

[4] Groen B.B., Res P.T., Pennings B., Hertle E., Senden J.M., Saris W.H., van Loon L.J. (2012). Intragastric protein administration stimulates overnight muscle protein synthesis in elderly men. Am. J. Physiol. Endocrinol. Metab..

[5] Koopman R., Walrand S., Beelen M., Gijsen A.P., Kies A.K., Boirie Y., Saris W.H., van Loon L.J. (2009). Dietary protein digestion and absorption rates and the subsequent postprandial muscle protein synthetic response do not differ between young and elderly men. J. Nutr..

[6] Pennings B., Boirie Y., Senden J.M., Gijsen A.P., Kuipers H., van Loon L.J. (2011). Whey protein stimulates postprandial muscle protein accretion more effectively than do casein and casein hydrolysate in older men. Am. J. Clin. Nutr..

[7] Tang J.E., Moore D.R., Kujbida G.W., Tarnopolsky M.A., Phillips S.M. (2009). Ingestion of whey hydrolysate, casein, or soy protein isolate: effects on mixed muscle protein synthesis at rest and following resistance exercise in young men. J. Appl. Physiol..

[8] Giezenaar C., Hutchison A.T., Luscombe-Marsh N.D., Chapman I., Horowitz M., Soenen S. (2017). Effect of age on blood glucose and plasma insulin, glucagon, ghrelin, CCK, GIP, and GLP-1 responses to whey protein ingestion. Nutrients.

[9] Giezenaar C., Trahair L.G., Rigda R., Hutchison A.T., Feinle-Bisset C., Luscombe-Marsh N.D., Hausken T., Jones K.L., Horowitz M., Chapman I. (2015). Lesser suppression of energy intake by orally ingested whey protein in healthy older men compared with young controls. Am. J. Physiol. Regul. Integr. Comp. Physiol..

[10] Steinert R.E., Feinle-Bisset C., Asarian L., Horowitz M., Beglinger C., Geary N. (2017). Ghrelin, CCK, GLP-1, and PYY(3–36): secretory controls and physiological roles in eating and glycemia in health, obesity, and after RYGB. Physiol. Rev..

[11] Soenen S., Giezenaar C., Hutchison A.T., Horowitz M., Chapman I., Luscombe-Marsh N.D. (2014). Effects of intraduodenal protein on appetite, energy intake, and antropyloroduodenal motility in healthy older compared with young men in a randomized trial. Am. J. Clin. Nutr..

[12] Rolls B.J., Dimeo K.A., Shide D.J. (1995). Age-related impairments in the regulation of food intake. Am. J. Clin. Nutr..

[13] Roberts S.B., Fuss P., Heyman M.B., Evans W.J., Tsay R., Rasmussen H., Fiatarone M., Cortiella J., Dallal G.E., Young V.R. (1994). Control of food intake in older men. JAMA.

[14] Blom W.A., Lluch A., Stafleu A., Vinoy S., Holst J.J., Schaafsma G., Hendriks H.F. (2006). Effect of a high-protein breakfast on the postprandial ghrelin response. Am. J. Clin. Nutr..

[15] Goetze O., Steingoetter A., Menne D., van der Voort I.R., Kwiatek M.A., Boesiger P., Weishaupt D., Thumshirn M., Fried M., Schwizer W. (2007). The effect of macronutrients on gastric volume responses and gastric emptying in humans: a magnetic resonance imaging study. Am. J. Physiol. Gastrointest. Liver Physiol..

[16] Giezenaar C., Lange K., Hausken T., Jones K.L., Horowitz M., Chapman I., Soenen S. (2018). Acute Effects of Substitution, and Addition, of Carbohydrates and Fat to Protein on Gastric Emptying, Blood Glucose, Gut Hormones, Appetite, and Energy Intake. Nutrients.

[17] Giezenaar C., van der Burgh Y., Lange K., Hatzinikolas S., Hausken T., Jones K.L., Horowitz M., Chapman I., Soenen S. (2018). Effects of substitution, and adding of carbohydrate and fat to whey-protein on energy intake, appetite, gastric emptying, glucose, insulin, ghrelin, CCK and GLP-1 in healthy older men-a randomized controlled trial. Nutrients.

[18] Gentilcore D., Hausken T., Horowitz M., Jones K.L. (2006). Measurements of gastric emptying of low- and high-nutrient liquids using 3D ultrasonography and scintigraphy in healthy subjects. Neurogastroenterol. Motil..

[19] Parker B.A., Ludher A.K., Loon T.K., Horowitz M., Chapman I.M. (2004). Relationships of ratings of appetite to food intake in healthy older men and women. Appetite.

[20] Matthews D.R., Hosker J.P., Rudenski A.S., Naylor B.A., Treacher D.F., Turner R.C. (1985). Homeostasis model assessment: insulin resistance and β-cell function from fasting plasma glucose and insulin concentrations in man. Diabetologia.

[21] Bland J.M., Altman D.G. (1995). Calculating correlation coefficients with repeated observations: part 1-correlation within subjects. Br. Med. J..

[22] Giezenaar C., Chapman I., Luscombe-Marsh N., Feinle-Bisset C., Horowitz M., Soenen S. (2016). Ageing is associated with decreases in appetite and energy intake--a meta-analysis in healthy adults. Nutrients.

[23] Rayner C.K., MacIntosh C.G., Chapman I.M., Morley J.E., Horowitz M. (2000). Effects of age on proximal gastric motor and sensory function. Scand. J. Gastroenterol..

[24] Soenen S., Hochstenbach-Waelen A., Westerterp-Plantenga M.S. (2011). Efficacy of alpha-lactalbumin and milk protein on weight loss and body composition during energy restriction. Obesity (Silver Spring).

[25] Weigle D.S., Breen P.A., Matthys C.C., Callahan H.S., Meeuws K.E., Burden V.R., Purnell J.Q. (2005). A high-protein diet induces sustained reductions in appetite, ad libitum caloric intake, and body weight despite compensatory changes in diurnal plasma leptin and ghrelin concentrations. Am. J. Clin. Nutr..

[26] Soenen S., Westerterp-Plantenga M.S. (2008). Proteins and satiety: implications for weight management. Curr. Opin. Clin. Nutr. Metab. Care.

[27] Pennings B., Groen B., de Lange A., Gijsen A.P., Zorenc A.H., Senden J.M., van Loon L.J. (2012). Amino acid absorption and subsequent muscle protein accretion following graded intakes of whey protein in elderly men. Am. J. Physiol. Endocrinol. Metab..

[28] Sturm K., MacIntosh C.G., Parker B.A., Wishart J., Horowitz M., Chapman I.M. (2003). Appetite, food intake, and plasma concentrations of cholecystokinin, ghrelin, and other gastrointestinal hormones in undernourished older women and well-nourished young and older women. J. Clin. Endocrinol. Metab..

[29] Camilleri M. (2006). Integrated upper gastrointestinal response to food intake. Gastroenterology.

[30] Horowitz M., Maddern G.J., Chatterton B.E., Collins P.J., Harding P.E., Shearman D.J. (1984). Changes in gastric emptying rates with age. Clin. Sci. (Lond).

[31] Soenen S., Rayner C.K., Horowitz M., Jones K.L. (2015). Gastric emptying in the elderly. Clin. Geriatr. Med..

[32] Pham H., Phillips L., Trahair L., Hatzinikolas S., Horowitz M., Jones K.L. (2020). Longitudinal changes in the blood pressure responses to, and gastric emptying of, an oral glucose load in healthy older subjects. J. Gerontol. A Biol. Sci. Med. Sci..

[33] Nauck M.A., Niedereichholz U., Ettler R., Holst J.J., Orskov C., Ritzel R., Schmiegel W.H. (1997). Glucagon-like peptide 1 inhibition of gastric emptying outweighs its insulinotropic effects in healthy humans. Am. J. Physiol..

[34] MacIntosh C.G., Horowitz M., Verhagen M.A., Smout A.J., Wishart J., Morris H., Goble E., Morley J.E., Chapman I.M. (2001). Effect of small intestinal nutrient infusion on appetite, gastrointestinal hormone release, and gastric myoelectrical activity in young and older men. Am. J. Gastroenterol..

[35] Trahair L.G., Horowitz M., Rayner C.K., Gentilcore D., Lange K., Wishart J.M., Jones K.L. (2012). Comparative effects of variations in duodenal glucose load on glycemic, insulinemic, and incretin responses in healthy young and older subjects. J. Clin. Endocrinol. Metab..

[36] Cowie C.C., Rust K.F., Byrd-Holt D.D., Gregg E.W., Ford E.S., Geiss L.S., Bainbridge K.E., Fradkin J.E. (2010). Prevalence of Diabetes and High Risk for Diabetes Using A1C Criteria in the U.S. Population in 1988–2006. Diabetes Care.

[37] Lee C.G., Boyko E.J., Strotmeyer E.S., Lewis C.E., Cawthon P.M., Hoffman A.R., Everson-Rose S.A., Barrett-Connor E., Orwoll E.S., Osteoporotic Fractures in Men Study Research Group (2011). Association Between Insulin Resistance and Lean Mass Loss and Fat Mass Gain in Older Men without Diabetes Mellitus. J. Am. Geriatr. Soc..

[38] MacIntosh C.G., Andrews J.M., Jones K.L., Wishart J.M., Morris H.A., Jansen J.B., Morley J.E., Horowitz M., Chapman I.M. (1999). Effects of age on concentrations of plasma cholecystokinin, glucagon-like peptide 1, and peptide YY and their relation to appetite and pyloric motility. Am. J. Clin. Nutr..

[39] Murphy K.G., Bloom S.R. (2004). Gut hormones in the control of appetite. Exp. Physiol..

[40] MacIntosh C.G., Morley J.E., Wishart J., Morris H., Jansen J.B., Horowitz M., Chapman I.M. (2001). Effect of exogenous cholecystokinin (CCK)-8 on food intake and plasma CCK, leptin, and insulin concentrations in older and young adults: evidence for increased CCK activity as a cause of the anorexia of aging. J. Clin. Endocrinol. Metab..

[41] Ranganath L., Sedgwick I., Morgan L., Wright J., Marks V. (1998). The ageing entero-insular axis. Diabetologia.

[42] Di Francesco V., Barazzoni R., Bissoli L., Fantin F., Rizzotti P., Residori L., Antonioli A., Graziani M.S., Zanetti M., Bosello O. (2010). The quantity of meal fat influences the profile of postprandial hormones as well as hunger sensation in healthy elderly people. J. Am. Med. Dir. Assoc..

[43] Pham H., Marathe C.S., Phillips L.K., Trahair L.G., Hatzinikolas S., Huynh L., Wu T., Nauck M.A., Rayner C.K., Horowitz M. (2019). Longitudinal Changes in Fasting and Glucose-Stimulated GLP-1 and GIP in Healthy Older Subjects. J. Clin. Endocrinol. Metab..

[44] Hall W.L., Millward D.J., Long S.J., Morgan L.M. (2003). Casein and whey exert different effects on plasma amino acid profiles, gastrointestinal hormone secretion and appetite. Br. J. Nutr..

[45] Giezenaar C., Luscombe-Marsh N.D., Hutchison A.T., Lange K., Hausken T., Jones K.L., Horowitz M., Chapman I., Soenen S. (2018). Effect of gender on the acute effects of whey protein ingestion on energy intake, appetite, gastric emptying and gut hormone responses in healthy young adults. Nutr. Diabetes.

[46] Giezenaar C., Trahair L.G., Luscombe-Marsh N.D., Hausken T., Standfield S., Jones K.L., Lange K., Horowitz M., Chapman I., Soenen S. (2017). Effects of randomized whey-protein loads on energy intake, appetite, gastric emptying, and plasma gut-hormone concentrations in older men and women. Am. J. Clin. Nutr..

