# Supplementary figures and images for: Effects of Age on Acute Appetite-Related Responses to Whey-Protein Drinks, Including Energy Intake, Gastric Emptying, Blood Glucose, and Plasma Gut Hormone Concentrations—A Randomized Controlled Trial
Source: Nutrients. 2020 Apr 6;12(4):1008. doi: 10.3390/nu12041008 (PMC7231005; doi:10.3390/nu12041008)

## CONSORT 2010 Flow Diagram

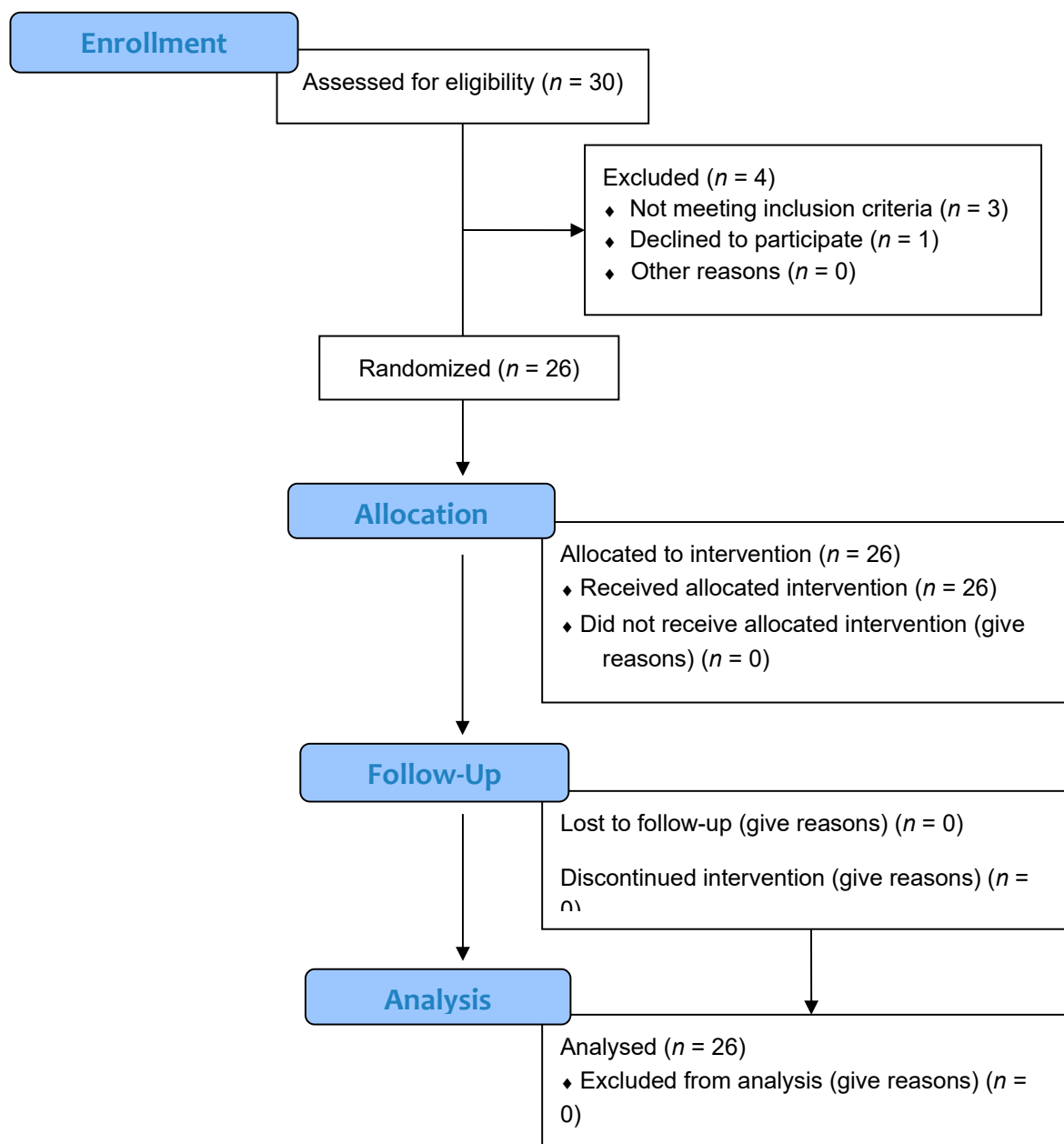

Supplement: Supplementary file 1 [file nutrients-12-01008-s001.pdf]
